# Hospital and Institutional News

**Published:** 1921-02-26

**Authors:** 


### HOSPITAL AND INSTITUTIONAL NEWS.

### THE DUKE OF YORK AND STATE SUPPORT.

The annual dinner of the Hospital Saturday Fund was a most successful affair, and the feature of the evening was the Duke of York's speech. He said that the success of the Fund's efforts was a source of great satisfaction to the King. The part of the Duke's speech that attracted special attention was that which referred to the possibility of State support supplementing voluntary contributions, and of central organisation—such as the Hospital Saturday Fund, King Edward's Hospital Fund, and the Sunday Fund—acting as the almoners of such augmented revenue. Coming from such a high quarter this sentiment has given much food for thought, as the elective features of the Sunday and Saturday Funds differ from the King's Fund, and before such a scheme can enter the sphere of practical politics ample opportunity for discussion by all concerned must be afforded. We are glad to know that the Duke of York does not consider that State aid necessarily means State control. The bedside manner, he said, of a cold and rigid bureaucracy might cure suffering humanity efficiently, but it is difficult to imagine it curing sympathetically. To his mind the pressing need is to preserve the kindly atmosphere and elastic methods of the voluntary system.

### THE DANGEROUS DRUGS REGULATIONS.

Daily there appears to be more and more agitation against the Dangerous Drugs Regulations of the Home Office. The British Medical Association and its branches have, in the volume of their protests, already outdone the initial outcry coming from pharmaceutical quarters. Now the veterinary surgeons find that the legitimate use of an important series of drugs will be seriously handicapped. We are getting used nowadays to these storms of protest against measures affecting the hospital spheres, but in this instance there does seem to be just cause for reconsideration, when the practical and not the ideal workings of the regulations are kept strictly in mind.

### THE CONTROL OF VACCINES AND SERA.

The Committee appointed by the Minister of Health to advise what measures should be taken to ensure the effective control of the quality and authenticity of sera, vaccines, salvarsan, and certain other medical agents which cannot be adequately tested by direct chemical means have completed their report. It is hoped that the report of the Committee, which contains matter of great interest to commercial firms manufacturing or dealing in these substances, as well as the general public, will shortly be published. The inquiries of the Committee must not be confused with those of the Select Committee on Patent Medicines which reported in 1913. The work of the present Committee is neither a duplication nor a continuation of that work, but deals with an entirely separate problem.

### THE RECORD CARDS.

In view of the vigorous discussions which have raged round the new medical record cards and more recently centred upon their possible production in courts of law, it is satisfactory to read that the Committee of medical men in the House of Commons met on February 21, with Lieutenant-Colonel Raw in the chair, when two resolutions were adopted. The first set out that the principle of keeping a record was likely to result in permanent advantage to the insured person; the second affirmed that the most jealous regard should be maintained for professional secrecy, which ought not to be infringed. It was decided that a question should be addressed to Dr. Addison with a view to ascertaining whether the records could be called for in a court of law.

### THE PLIGHT OF A KEY INDUSTRY.

We trust that due public attention will be given to the letter now circulated by the Association of British Chemical Manufacturers, as representing one of the most important key industries in the country. Research and manufacturing plants were extended at the urgent appeal of the State during the tremendous years that followed 1914, in order to secure those essential products of science without which victory could not be attained. This industry is now undoubtedly endangered. Unemployment is rife in it, owing to the flood of imports from abroad which, among other circumstances, the present condition of the exchanges makes devastatingly possible. We feel it should undoubtedly be made plain to the public that here is an industry of such national importance that in no circumstances must it be omitted from whatever safeguards for key industries the State intends in the near future to provide.

### RED CROSS WAR FINANCE.

The accounts of the Joint War Finance Committee of the British Red Cross Society and the Order of St. John from October 20, 1918, to June 30, 1920, have been issued, together with a final report on the financial operations of the Committee and a summary of accounts from October 20, 1914, to June 30, 1920. The latter will be included in a detailed record of the whole war work of the Joint Organisation which has been in preparation since the Armistice, and will be published shortly. We hope the next volume will include the accounts of Our Day, 1920, which are looked forward to with interest. The present volume can be obtained from the Inquiry Officer, 19 Berkeley Street, W. 1., for 1s. It will be remembered that the Joint Organisation of the two Corporations, set up for the purposes of the war, was placed under the financial control of a Finance Committee which was presided over by Sir Robert Hudson, G.B.E., and that annual accounts have been rendered to the public throughout the war, which we have reviewed from time to time. Some idea of the magnitude of the financial work which fell to this Committee will be gained from the fact that they have had to account for receipts amounting to £21,885,035, and expenditure £20,058,355. Of the receipts £16,510,023 were received in money from the public and £1,027,280 in kind; Government grants, interest, and various receipts from other bodies making up the total. The chief items of expenditure were £2,053,501 for the transport of wounded; £2,928,367 for hospitals, not including local expenditure on the Auxiliary Home hospitals; £5,017,285 for stores; £5,147,876 for prisoners of war, and £2,717,927 for post-war schemes for sick and disabled ex-Service men and kindred objects. These latter are provided for by the Red Cross and St. John Act of 1918 which governs the disposal of any surplus. The surplus on June 30 last amounted to £1,826,680, and the Committee in their report foreshadow the development of a scheme throughout the country for the benefit of disabled ex-Service men, the particulars of which will be awaited with much interest.

### A HOSPITAL SECRETARY'S ILLNESS.

We regret to learn of the illness of Mr. Wilson, Secretary of the Durham County Hospital. Apart from the temporary loss of his administrative services, Mr. Wilson's illness has interfered with the production of the annual report. All his friends will hope to learn of his speedy recovery.

### UNEMPLOYMENT AND WORKERS' CONTRIBUTIONS.

Many hospitals are suffering through the prevalent unemployment, which, in the usual disastrous way, succeeded the trade boom during the war. Collections diminish as men are discharged, and the assessment and collection of patients' payments have often become more than usually difficult. At the same time, it is probable that unemployment increases rather than diminishes the number of patients, and, if statistics can be gathered to prove this, the hospitals would be entitled to ask for assistance from the Ministry of Labour, in the same way as they have been assisted by the Ministry of Pensions for treatment of cases under its peculiar charge.

### THE BOILER EXPLOSION IN CITY ROAD.

The outcome of the coroner's inquiry into the cause of the death of two men through a boiler explosion at the Royal Chest Hospital was that the accident was caused by an error of judgment. Evidence was given that a night porter who stoked the boiler fires had found that the flange of the door of No. 2 boiler had fallen off, and he had lit the fire in No. 1 boiler, which he had no right to do.

### FRUITS OF CO-OPERATION IN BIRMINGHAM.

How far the Birmingham Guardians have already helped the voluntary hospitals can be judged from the following published figures: From April to December 1920, it is reported, the Dudley Road, Selly Oak, and Erdington Infirmaries received 432 cases from the hospitals, and 565 cases certified to be urgent. In fit cases contributions have been received from patients or their relatives. The full and interesting facts will be known when the report upon the work of the institutions shall be published. It is hoped to make the report available in April next.

### THE HEART OF LABOUR.

Everybody was shaking hands with himself and everybody else at Thursday's meeting of the Swansea Hospital Board. Mr. Roger Beck, the Chairman, gave a most encouraging report on the year's finances. The receipts were £20,000 up, being £55,621, as compared with £35,000 the previous year. "Labour," said the Chairman, "has not only done better than ever before, but better than was expected, and the heart of Labour has evidently warmed the heart of Capital, for the big donations have also increased." Then Mr. Lewis reported that the waiting-list is being satisfactorily reduced, and that when the opening of Parc Wern is considered the prospect is gratifying. Parc Wern is preparing for early occupation, and the furniture is being ordered. The announcement was also conveyed in a letter from the testator's lawyer that a sixty-acre farm in Pembrokeshire has been bequeathed to the hospital by the late Mr. Fisher, of Briton Ferry.

### THE PRESTIGE OF DENTAL HOSPITALS.

Hospital jubilees are apt to pass unnoticed unless they mark some new and important development. This is not always easy in respect of special hospitals, but the Liverpool Dental Hospital, which celebrates its diamond jubilee this year, is in an exceptionally favourable position. Of all the special hospitals, which still have their critics and zealous advocates, the dental hospitals have, perhaps, of late developed more than any. The once underrated importance of dental surgery is underrated no longer. Oral sepsis is now known even by the general public to be the source of much preventable disease. This recognition, abundantly supported by evidence in the war, has given to dental hospitals a new standing. The Liverpool Dental Hospital has several proofs of this. In the year of its jubilee it can boast a friend who has promised to double the amount of new subscriptions; an honorary secretary, in Mr. W. L. Jackson, who has worked for it for forty-three years; the foundation of the first chair of dentistry by the generosity of Alderman Cohen, and the affiliation of the hospital to the University of Liverpool. With these cards in his hand, Mr. Jackson should be able to crown his efforts by removing the deficit of £13,000, and to find the money for the new developments which are needed. We congratulate the Liverpool Dental Hospital on its leadership in these matters, which will enhance the respect given to all the dental hospitals.

### UNEXPLAINED FIRE AT A HOSPITAL.

Three separate outbreaks of fire occurred at the Warneford Hospital, Leamington, during the night of the 13th inst. One was in the house-surgeon's sitting-room, another in his office, and the third in the matron's store-room. Happily little damage was done, and what might have been a calamity was averted. The local fire-brigade was at once called, and there was no necessity to remove any of the patients from the institution. Fortunately, accidental fires of any extent in hospitals where there are people about night and day are very rare. Fires which are not accidental are rarer still, and if that which occurred at Leamington can be so described we trust that the person responsible will be detected and severely dealt with.

### PATIENTS AT EACH OTHER'S FUNERALS.

When submitting a series of minor alterations to the statutes of the Royal Hampshire County Hospital, Mr. Henry Nicholl assured the Governors that nothing so startling was now proposed as the alteration of the rule which was carried, as he remembered, about twenty-four years ago, on a similar occasion. They then revised an ancient rule whereby all patients who were sufficiently well were obliged to attend the funerals of their fellows. A surviving rule which he asked should be changed, however, was one which forbade expectant mothers to be admitted. Their recent maternity department was the living contradiction of the statute. He urged also that the rule, suspended during the war, that patients must not smoke in the male wards also be swept away. The alterations were approved, and a chapter of history was quietly closed with them.

### COTTAGE HOSPITALS JOIN THE MOVEMENT.

We observe with interest that Mr. F. D. Wise, honorary secretary of Ripon Cottage Hospital, has recorded a new scheme for organising weekly contributions from the workers employed in the different industries of the town. So far as we know, the Ripon Cottage Hospital has been among the first institutions of its class to adopt such a scheme, and, since similar schemes are now being arranged successfully in country districts, it is plain that weekly contributions are capable of adoption by hospitals of every size, however small.

### WEEKLY CONTRIBUTIONS VERSUS COLLECTING-BOXES.

The York Workpeople's Hospital Fund, which has raised over £5,000 in the past year for the County Hospital, has issued an interesting report. It is recorded that since Messrs. Rowntree & Co. acceded to the request of their staff to allow the cashier to deduct 1d. per week from wages, the employees' subscriptions rose to £1,084, in encouraging contrast to the sum of £384, obtained from the collecting-boxes, which have now been superseded. The new plan, in short, has already trebled the total on the initiative of the employees themselves. Is there anyone still in doubt concerning the value of organised sectional subscriptions?

### RESULT OF THE KENT EYE HOSPITAL INCOME SCHEME.

We can understand the interest aroused in the scheme for a Weekly Subscribers' Fund associated with the Kent County Ophthalmic Hospital at Maidstone. Indeed we learn officially that in consequence of the publication of the scheme of the fund some time ago in these columns the organising secretary received inquiries from hospitals all over England and even Scotland. The numerous interested inquirers, who have asked the hospital to give them the fruits of its experience, that similar schemes may be started elsewhere, now have the report on the progress of the fund for the year ending December 31 last. The growth of the fund can be recorded in three lines. In 1918 twelve firms subscribed £192; in 1919 184 firms subscribed £2,231; last year 392 firms subscribed £5,786. The authors of the scheme, of which Mr. Ernest Rumbelow is the organising secretary, attribute its success to four causes: That the workers are directly represented on the board; that each firm has a representative to look after the interests of its subscribers; to the activities of factory committees and their officers; and to the employers' help in the encouragement of collections and of meetings. It is noteworthy that the Dover Hospital Workers' Committee is thanked for its support, and that it has a representative on the board of the Kent County Ophthalmic Hospital. Thus the net seems to have spread widely, and yet with due regard for interests elsewhere.

### FIRE ESCAPE AT A MUNICIPAL LYING-IN HOSTEL.

Bermondsey Borough Council is trying to persuade the Ministry of Health to sanction adequate provisions at its lying-in hostel against an outbreak of fire. The Ministry maintain that in places of this kind, where the number of occupants does not exceed twenty, an outside fire-escape staircase is not required, but that an exit from the roof will suffice. The requirements of the London County Council cannot be modified, and, in order that the hostel shall be as safe as it is possible to make it, the Borough Council is pressing the Ministry to sanction the staircase. The hostel, one of the first municipal ones in London, is proving already a great boon to the mothers in the overcrowded borough, where it is often impossible to get proper care and attention in the one or two rooms often occupied by a large family of young children, in addition to their parents.

### THIS WEEK'S DRUG MARKET.

A fairly long list of drugs can be quoted at further reduced prices, and in some instances these are subject to negotiation, as buyers still require a good deal of temptation before they will commit themselves. Most of the synthetic products are obtainable at rather lower prices, and supplies from Continental sources are accumulating. The demand for opium has fallen off and prices are lower. Cream of tartar is substantially cheaper. Epsom salts and Glauber's salts are offered at low rates. Clove oil is quoted at reduced rates in consequence of the lower price of cloves. Ergot of rye is again cheaper. The camphor market continues very quiet, with prices tending downwards. Castor oil is slightly higher in price.

